# Anesthetic Management During Splenectomy for Severe Platelet Sequestration in a Human Immunodeficiency Virus (HIV)-Positive Patient: A Case Report

**DOI:** 10.7759/cureus.111318

**Published:** 2026-06-22

**Authors:** Sara Trujano de la Rosa, Claudia C Nando-Villicaña, Cristian I Ham-Armenta, Horacio Olivares Mendoza, Ana Cristina López Zepeda, Jessica Esquivel Chahin

**Affiliations:** 1 Anesthesiology, Hospital Angeles Lomas, Huixquilucan, MEX; 2 Anesthesia and Critical Care, Hospital Angeles Lomas, Huixquilucan, MEX

**Keywords:** burkitt lymphoma, general anesthesia, human immunodeficiency virus, regional analgesia, splenectomy, thrombocytopenia

## Abstract

Patients with compromised immune systems, particularly those living with human immunodeficiency virus (HIV) infection and HIV-related lymphoma, pose multiple challenges for anesthesiologists because of hematologic abnormalities, critically low platelet counts, and potential drug-drug interactions.

This report describes a 55-year-old male patient with HIV and Burkitt-type non-Hodgkin lymphoma (NHL) who presented with splenomegaly and severe platelet sequestration. Preoperative hematologic optimization was achieved with two platelet apheresis sessions and tranexamic acid for antifibrinolytic prophylaxis. The patient underwent an elective splenectomy under balanced general anesthesia. Propofol, fentanyl, and rocuronium were used for anesthetic induction, and tracheal intubation was successfully performed using videolaryngoscopy without complications. Following surgery, transversus abdominis plane (TAP) blocks and rectus sheath blocks were performed using ropivacaine, providing adequate analgesia without the need for postoperative opioids.

The patient remained hemodynamically stable throughout the procedure, with an uneventful anesthetic recovery and no bleeding or infectious complications. He was discharged on postoperative day 3, with a platelet count of 80,000/µL.

Optimal perioperative management of HIV-positive patients with severe thrombocytopenia is essential for safe anesthetic care. Appropriate blood product utilization and regional analgesia techniques may further improve outcomes. Multidisciplinary management is crucial for minimizing perioperative risks and enhancing patient outcomes.

## Introduction

Anesthetic management of patients with human immunodeficiency virus (HIV)-associated Burkitt lymphoma presents significant challenges because of the profound perioperative implications of severe thrombocytopenia and the presence of complex comorbidities. The primary anesthetic challenge lies in mitigating the extreme risk of bleeding, underscoring the need for thorough preoperative evaluation and a multidisciplinary approach [1,2], while taking into account the hematological, immunological, and pharmacological characteristics of these vulnerable patients. The coexistence of HIV infection and aggressive B-cell malignancies, such as Burkitt lymphoma, further increases surgical and anesthetic complexity [3-6].

Although splenectomy is often indicated in these patients to address platelet sequestration and must be performed under strict safety and anesthetic protocols, with careful consideration of hematologic and cardiovascular parameters [2], comprehensive perioperative management must recognize that the profound thrombocytopenia observed in HIV-associated lymphomas is fundamentally multifactorial. This condition results from a combination of splenic sequestration, immune-mediated destruction, direct viral infection of megakaryocytes, bone marrow involvement by the malignancy, and the myelosuppressive effects of chemotherapy [7-9].

Management of these patients requires more than standard perioperative care; it necessitates an individualized preoperative hematologic optimization strategy to achieve safe platelet thresholds, typically ≥40-50 × 10⁹/L for major abdominal procedures, together with effective perioperative analgesia through the use of specialized regional anesthesia techniques while minimizing bleeding risks [1]. There remains a significant gap in the literature regarding optimal standardized multidisciplinary protocols for these patients, and the feasibility of fascial plane regional anesthesia techniques, such as transversus abdominis plane (TAP) and rectus sheath blocks, in the setting of severe multifactorial thrombocytopenia remains poorly defined.

This case highlights the critical role of a coordinated multidisciplinary approach in optimizing care for a complex immunocompromised patient. It also demonstrates the feasibility of combining targeted hematologic resuscitation with opioid-sparing regional anesthesia to achieve a favorable surgical outcome.

## Case presentation

A 55-year-old man with HIV infection (stage C3), latent syphilis, and Burkitt-type B-cell non-Hodgkin lymphoma (NHL) (WHO 2022 classification) was evaluated. Immunophenotyping revealed CD20+, Bcl-6+, CD10+, and C-MYC+ expression, with a proliferation index of 95%. The lymphoma was associated with Epstein-Barr virus (EBER-1+). The patient was undergoing his third cycle of chemotherapy (R-CODOX/R-IVAC) and was receiving antiretroviral therapy with Biktarvy (bictegravir/emtricitabine/tenofovir alafenamide; one tablet orally every 24 hours), with good adherence.

During a follow-up consultation, complementary laboratory studies revealed significant thrombocytopenia. Clinically, the patient remained asymptomatic, with no evidence of hemorrhagic or purpuric manifestations secondary to thrombocytopenia. However, the condition resulted in functional limitations, predisposing him to complications such as sarcopenia, generalized weakness, impaired secretion clearance, and the inability to continue lymphoma treatment.

According to previous evaluations by the Hematology service, the patient had persistent multifactorial thrombocytopenia (secondary to splenomegaly and an immune-mediated component) that was refractory to intravenous immunoglobulin (IVIG), corticosteroids, and thrombopoietin receptor agonists (TPO-RAs). Based on this clinical course, the history of recurrent infections, and previous laboratory findings, laparoscopic splenectomy was indicated to manage splenic platelet sequestration after intrinsic thrombopoietin deficiency had been excluded. Immunization against encapsulated organisms, including *Streptococcus pneumoniae* and *Neisseria meningitidis*, was administered 15 days before the procedure.

To complement the perioperative risk assessment associated with HIV infection, data from a previous molecular biology report obtained six months earlier were reviewed. Quantitative real-time polymerase chain reaction (PCR) performed on total RNA isolated from plasma revealed an HIV-1 viral load of 2,540,000 copies/mL (4,368,800 IU/mL). Concurrently, lymphocyte subset analysis by flow cytometry demonstrated the following profile: CD3+ T lymphocytes, 73.4% (absolute count, 1,459 cells/µL); CD4+CD3+ T lymphocytes, 6.0% (absolute count, 119 cells/µL); and CD8+CD3+ T lymphocytes, 65.4% (absolute count, 1,300 cells/µL). The most recent viral load measurement (September 2024) was 71 copies/mL.

**Figure 1:**
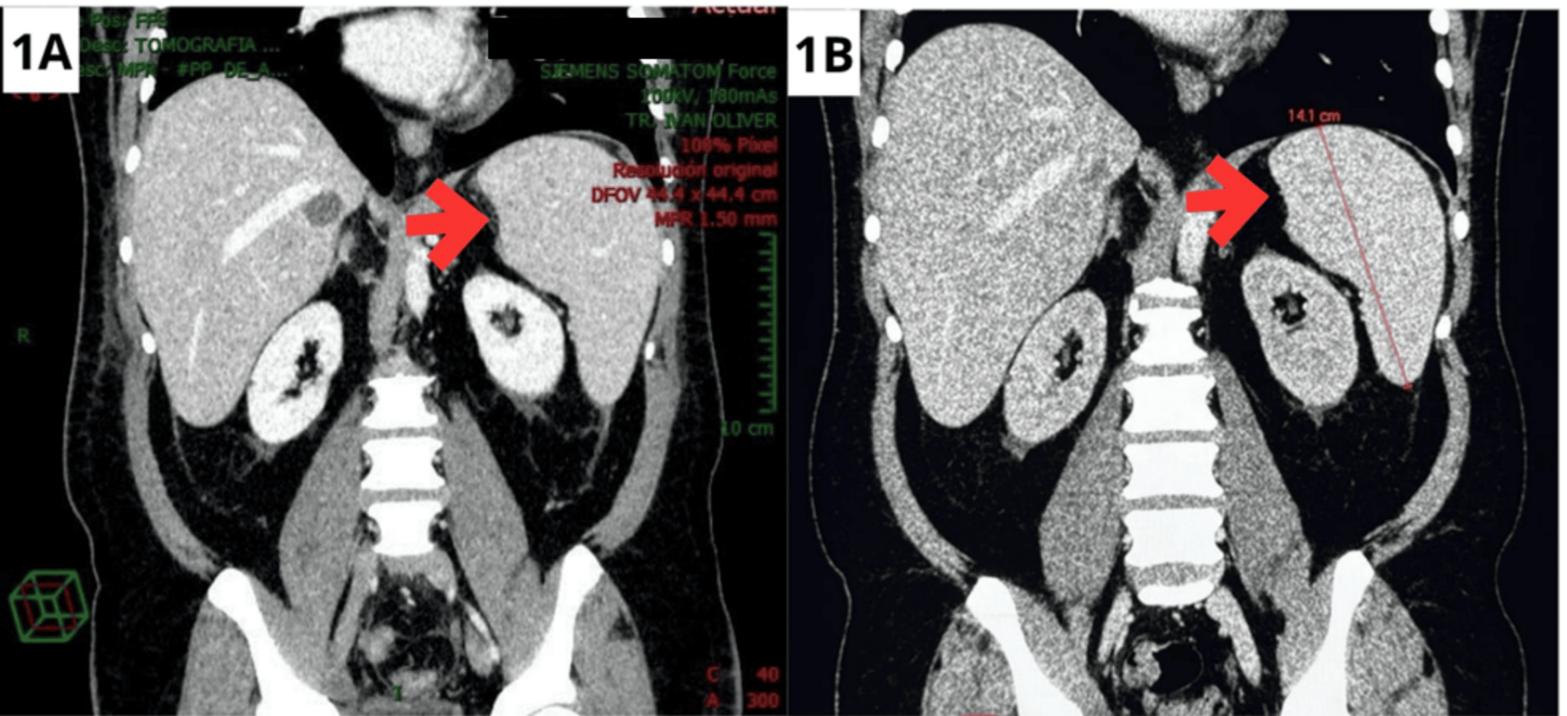
Abdominal contrast-enhanced computed tomography (CECT) coronal multiplanar reconstruction (MPR) obtained during the portal venous phase (A) Coronal image obtained at the initial evaluation demonstrating severe splenomegaly, with a calculated splenic volume of 605.0 cc (arrow). (B) Follow-up coronal image obtained six months later demonstrating progression of splenomegaly, with an increased splenic volume of 631.6 cc (arrow). The images highlight the significant mass effect exerted on adjacent abdominal structures.

The patient was admitted because of splenomegaly with severe platelet sequestration. Admission laboratory tests revealed hemoglobin of 10.36 g/dL, hematocrit of 32.9%, platelet count of 11 × 10³/µL, and white blood cell count of 2.82 × 10³/µL (absolute neutrophil count (NEUABS), 1.30 × 10³/µL; neutrophils, 46.0%). Renal function tests showed urea of 49.6 mg/dL and creatinine of 0.81 mg/dL. Liver function tests demonstrated albumin of 3.34 g/dL, total bilirubin of 0.41 mg/dL, direct bilirubin of 0.22 mg/dL, indirect bilirubin of 0.19 mg/dL, aspartate aminotransferase (AST) of 50.2 U/L, alanine aminotransferase (ALT) of 85.9 U/L, alkaline phosphatase (ALP) of 126.8 U/L, lactate dehydrogenase (LDH) of 207.0 U/L, and gamma-glutamyl transferase (GGT) of 447.3 U/L. Coagulation studies showed normal values: prothrombin time (PT), 10.3 s; international normalized ratio (INR), 0.85; activated partial thromboplastin time (aPTT), 31.4 s; and thrombin time (TT), 12.1 s. C-reactive protein (CRP) was 0.700 mg/dL. In addition, cystatin C was 1.50 mg/L, with an estimated glomerular filtration rate (eGFR) of 50.69 mL/min/1.73 m². Initial abdominal computed tomography demonstrated hepatosplenomegaly (Figure 1).

Past surgical history included hepatic biopsy and segmentectomy (June 2024) and port-a-cath placement (June 2024), both of which were performed without anesthetic complications.

Pre-anesthetic evaluation classified the patient as ASA physical status III and RAQ EIIIB. Cardiac risk was classified as Revised Cardiac Risk Index (RCRI) class II, corresponding to a 6.0% risk, while pulmonary risk was considered high according to the ARISCAT score (42 points). No predictors of a difficult airway were identified. Two units of platelet apheresis were transfused six hours before surgery, safely increasing the platelet count to 60 × 10⁹/L. Premedication consisted of tranexamic acid 1 g intravenously administered 30 minutes before surgical incision. Preoxygenation was performed via face mask at 6 L/min. Anesthesia was induced with propofol 130 mg, fentanyl 200 µg, and rocuronium 50 mg. Videolaryngoscopy using a C-MAC device achieved a Percentage of Glottic Opening (POGO) score of 100%, and an 8.0-mm endotracheal tube was placed uneventfully. Anesthesia was maintained with sevoflurane at 2 vol% (1 minimum alveolar concentration (MAC)). Mechanical ventilation was provided using a volume-controlled mode.

The intraoperative course remained hemodynamically stable. Mean arterial pressure (MAP) was maintained within a perfusion range of 60-70 mmHg, and no vasopressor support was required. Estimated blood loss was 50 mL, total crystalloid administration was 1,100 mL, urine output was 1.0 mL/kg/h, and the duration of surgery was 160 minutes. Although the preoperative optimization strategy with blood product and platelet transfusions improved the patient's clinical and laboratory status sufficiently to allow surgery, preoperative values remained below ideal thresholds. Therefore, to mitigate perioperative risks, maintain surgical hemostasis, and address a preoperative hemoglobin level of 8.85 g/dL, intraoperative transfusion therapy was continued. During splenic artery clamping, the patient received two additional units of platelet apheresis and one unit of packed red blood cells without adverse events (Table 1).

**Table 1:** Laboratory results

Parameter	Admission	Pre-op	Post-op	Discharge (post-op day 3)	Reference range
Hemoglobin (g/dL)	10.36	8.85	12.04	9.31	14.00-18.00
Hematocrit (%)	32.9	28.7	36.7	29.1	42.0-54.0
Platelets (10⁹/L)	11	59	126	80	130-450
White blood cell (10⁹/L)	2.82	1.84	3.18	9.13	3.80-11.20

At the end of the procedure, bilateral ultrasound-guided TAP and rectus sheath blocks were performed under sterile conditions using a linear ultrasound probe and a 100-mm Stimuplex needle. Ropivacaine 0.375% (15 mL per side) was administered bilaterally in the triangle of Petit, followed by ropivacaine 0.2% (10 mL per side) injected bilaterally into the rectus sheath.

Emergence from anesthesia was achieved with pharmacologic reversal using sugammadex 200 mg. The endotracheal tube was removed uneventfully, and the patient was transferred to the post-anesthesia care unit (PACU) awake, alert, and cooperative (Aldrete score, 9/10; Ramsay score, II), with stable hemodynamic parameters and a visual analog scale (VAS) pain score of 2/10. He was subsequently transferred to the hospital ward and did not require opioid rescue during his hospital stay. The patient was discharged on postoperative day 3 in good clinical condition, with a platelet count of 80 × 10⁹/L. No puncture-site bleeding, abdominal wall hematoma, or delayed block-related complications were observed during postoperative follow-up.

## Discussion

HIV infection has transformed the global health landscape, evolving from a disease associated with high morbidity and mortality into a treatable chronic condition [3,4]. Despite major therapeutic advances, malignant neoplasms remain a significant concern in this population. NHLs constitute the predominant group of HIV-associated malignancies, with Burkitt lymphoma and diffuse large B-cell lymphoma (DLBCL) accounting for approximately 90% of cases [4,5].

The pathogenesis of thrombocytopenia in HIV-positive patients is multifactorial and extends beyond simple splenic sequestration [6-8]. Although the spleen can sequester up to 33% of circulating platelets, contributing to the hypersplenism observed in the present case [2], thrombocytopenia is frequently exacerbated by immune-mediated platelet destruction and reduced platelet production secondary to direct HIV infection of megakaryocytes [8]. In patients with concomitant malignancies such as Burkitt lymphoma [4,6], these mechanisms may be further compounded by bone marrow involvement and the myelosuppressive effects of chemotherapy [3], necessitating a comprehensive hematologic evaluation [7,9-11].

Burkitt lymphoma is one of the most aggressive B-cell neoplasms, particularly in patients co-infected with Epstein-Barr virus (EBV), which is identified in 30%-40% of cases [6]. It is characterized by a high proliferative index and rapid clinical progression driven by immune dysregulation and uncontrolled B-cell activation. Extranodal involvement is common, frequently affecting the gastrointestinal tract and bone marrow [6], although involvement of the oral cavity is also well recognized [4]. Splenic involvement may manifest as splenomegaly or focal splenic masses [7], whereas hepatic involvement may present as parenchymal nodules, as described in hepatosplenic cystic-nodular Burkitt lymphoma [5]. In primary splenic DLBCL, imaging typically demonstrates hypodense lesions on contrast-enhanced computed tomography or hypoechoic lesions on ultrasonography.

HIV infection impairs hematopoiesis, predisposing patients to cytopenias such as anemia, leukopenia, and particularly thrombocytopenia, defined as a platelet count <150 × 10⁹/L [1]. The pathogenesis of thrombocytopenia is multifactorial and may involve immune-mediated platelet destruction, reduced platelet production, direct infection of megakaryocytes, and splenic sequestration [8]. The spleen plays a central role by sequestering up to 33% of circulating platelets [9], thereby promoting hypersplenism and cytopenias. In the perioperative setting, severe thrombocytopenia substantially increases bleeding risk and necessitates individualized transfusion strategies together with close intraoperative monitoring [4]. In stable, non-bleeding, non-surgical patients, prophylactic platelet transfusion is generally recommended when platelet counts are <10 × 10⁹/L [3,10]. However, for patients undergoing major surgery, current guidelines recommend a perioperative platelet threshold of ≥40-50 × 10⁹/L, as reflected in ASCO guidance. In the present case, a preoperative platelet count of ≥50 × 10⁹/L was successfully achieved [11].

Perioperative management of these patients is often highly complex. In elective surgery, a detailed preoperative evaluation is essential, and continuation of antiretroviral therapy (ART) is recommended whenever feasible [12]. Drug-drug interactions can pose significant challenges, particularly in patients undergoing abdominal surgery. Regarding anesthetic management, available evidence suggests that propofol and sevoflurane have comparable effects on inflammatory responses, and the choice of anesthetic agent should therefore be individualized according to the patient's clinical profile [13].

During surgery, maintaining hemodynamic stability and correcting thrombocytopenia are essential to ensure patient safety. In stable, non-bleeding patients, prophylactic platelet transfusion is generally recommended for platelet counts <10-20 × 10⁹/L, while restrictive red blood cell transfusion strategies (hemoglobin < 7 g/dL) are commonly advocated [10]. Antifibrinolytic agents such as tranexamic acid may reduce blood loss without significantly increasing thrombotic risk. In addition, advanced hemodynamic monitoring, thromboelastography, and goal-directed transfusion protocols may decrease the risk of hemorrhagic and thromboembolic complications.

Multimodal analgesia is a cornerstone of perioperative care. Regional anesthesia techniques, particularly fascial plane blocks such as transversus abdominis plane (TAP) blocks, provide effective analgesia and reduce opioid requirements [14]. Nevertheless, although ultrasound guidance decreases the incidence of complications, the risk of bleeding remains inherent to both neuraxial and peripheral regional anesthesia techniques. Consequently, meticulous case-by-case risk-benefit assessment and individualized decision-making remain mandatory, especially when deeper regional blocks are considered. In addition, adjuvants such as dexmedetomidine and dexamethasone may prolong analgesia and further reduce opioid consumption [15]. Local anesthetics and selective cyclooxygenase-2 (COX-2) inhibitors are generally considered safe [6], whereas non-selective nonsteroidal anti-inflammatory drugs (NSAIDs) should be avoided because of their potential to increase bleeding risk [16].

Prophylaxis against opportunistic infections and preoperative vaccination are essential in splenectomized and immunocompromised patients. Postoperative surveillance should focus on hemorrhage, infection, and neurological dysfunction. Both thrombocytopenia and splenectomy increase the risk of sepsis and other major complications, necessitating close clinical monitoring and comprehensive patient education.

## Conclusions

Anesthetic management of immunocompromised patients with HIV infection remains a complex challenge due to immunosuppression, hematologic disorders, and drug interactions. In this specific case, a multidisciplinary approach involving anesthesiologists, surgeons, infectious disease specialists, and hematologists made the perioperative care and surgical intervention feasible. Specifically, this case demonstrates that splenectomy was performed without early reported bleeding or infectious complications following careful platelet correction and multidisciplinary planning. Despite advances in anesthetic management, controversies remain regarding optimal transfusion thresholds and the role of regional anesthesia in severe thrombocytopenia. Further multicenter randomized trials are needed to establish evidence-based guidelines for this vulnerable population.

This study is a single case report, which inherently limits the generalization of these anesthetic management outcomes to a broader population. While the multidisciplinary strategy and the use of fascial-plane blocks proved feasible in this patient, this report does not establish the universal safety of regional anesthesia in patients with severe thrombocytopenia, nor does it definitively establish optimal transfusion thresholds. Furthermore, this report lacks long-term follow-up data beyond the immediate postoperative period to assess late complications or long-term safety of the multimodal strategies employed.
